# BST2 and DIRAS3 Drive Immune Evasion and Tumor Progression in High-Grade Glioma

**DOI:** 10.3390/ijms26136205

**Published:** 2025-06-27

**Authors:** Zhangjun Liao, Shuyi Wu, Zhenyi Shi, Donghui Chen, Jinrui Chen, Hua Zhang

**Affiliations:** Guangdong Provincial Key Laboratory of Medical Immunology and Molecular Diagnostics, School of Medical Technology, Guangdong Medical University, Dongguan 523808, China; lzj440982@163.com (Z.L.); wsy_cathrynwoo@outlook.com (S.W.); szysmu0128@163.com (Z.S.); dhchen_re@outlook.com (D.C.); cjr20020302@outlook.com (J.C.)

**Keywords:** high-grade gliomas, cytotoxic T lymphocytes, prognostic model, BST2, DIRAS3

## Abstract

High-grade gliomas (HGGs, WHO grades 3–4) are highly aggressive, with a poor prognosis and treatment resistance. Immune evasion may contribute to their progression, but the role of cytotoxic T-lymphocyte immune evasion (CTLE) is not well-validated. This study analyzed the transcriptomic data of 525 patients from TCGA-GBM-HG_U133A. Two molecular subtypes were identified based on 182 CTLE-associated genes, with 238 differentially expressed genes between them. A prognostic model was developed, identifying BST2 and DIRAS3 as key risk factors, and validated in multiple cohorts. The subtypes had distinct immune profiles, with Cluster 2 showing higher immune infiltration but a poorer prognosis. The model had a good predictive performance. High-risk patients had upregulated BST2 and DIRAS3, linked to immunosuppression and shorter survival. Knockdown experiments confirmed their roles in GBM cell migration and invasion. Mechanistically, they promote immune evasion. BST2 and DIRAS3 could be therapeutic targets for HGG immunotherapy.

## 1. Introduction

High-grade gliomas (HGGs, WHO grades 3–4) are highly aggressive malignant tumors of the central nervous system. Among these, WHO grade-4 gliomas (HGG-4s) are the most aggressive and have the worst prognosis [1]. HGG-4s are molecularly defined as two distinct entities: glioblastoma, IDH-wildtype (GBM); and astrocytoma, IDH-mutant. These tumors collectively account for 49% of primary malignant brain tumors and exhibit poor prognoses [2,3]. Despite multimodal therapy (maximal resection, radiotherapy, and temozolomide chemotherapy), HGG-4s exhibit an extremely poor prognosis and near-universal recurrence due to intrinsic radio–chemoresistance [2,3,4,5,6,7].

Current aggressive treatments, including surgical resection and radiotherapy combined with chemotherapy, still have a poor prognosis for recurrence-prone HGG-4s, with a median survival of 15–30 months [5,7].

Recently, targeted therapies and immunotherapies for gliomas have received much attention, but no effective immunotherapies for HGGs have been available to date [8,9]. However, emerging evidence suggests that glioblastomas may be particularly adept at evading the immune system, and the results of clinical trials targeting them in their early stages have been disappointing [10,11]. Consequently, there is an imperative necessity to identify novel molecular targets or prognostic indicators for the diagnosis and therapy of HGG-4s.

Following bioinformatics research and a review of the literature, BST2 (bone marrow stromal cell antigen 2) and DIRAS3 (DIRAS family GTPase 3, also referred to as absence of Ras homolog I (ARHI) and normal ovarian epithelium Y2 (NOEY2)) were chosen for comprehensive investigation.

BST2, also known as fasciculin, CD317, and HM1.24, is an antiviral protein. BST2, a type II transmembrane protein, has been demonstrated to have an oncogenic function in multiple myeloma, breast, lung, and colorectal cancers [12,13,14,15].

DIRAS3 is the first endogenous, non-RAS protein to heterodimerize with RAS, disrupt RAS clustering, block RAS signaling, and inhibit cancer cell growth [16]. DIRAS3 is a family of proto-oncogenic RASs, including KRAS, NRAS, and HRAS, that are GTPases localized to cell membranes that regulate cell proliferation, motility, and apoptosis [17]. The downregulation of DIRAS3 is seen in cancers of the ovary, breast, lung, prostate, colon, brain, and thyroid [17,18].

This study demonstrates the significant involvement of BST2 and DIRAS3 in enhancing HGG cell proliferation and migration, while also providing evidence of a relationship between HGG cells and T-cell immune evasion.

## 2. Results

### 2.1. Glioma Subtypes Based on Transcriptional Signatures Associated with Cytotoxic T-Lymphocyte-Mediated Immune Escape

**Figure 1:**
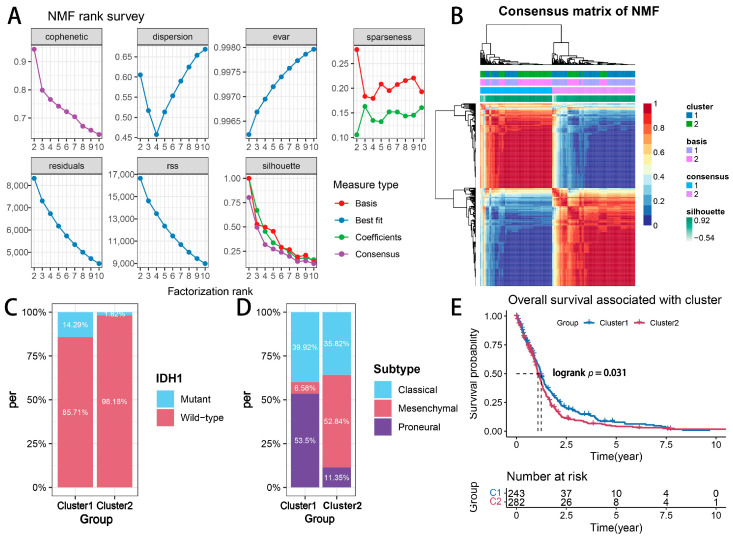
The identification of glioma subtypes based on transcriptional signatures associated with cytotoxic T-lymphocyte-mediated immune escape. (**A**) An NMF rank survey showing the optimal number of clusters (k = 2) based on the cophenetic coefficient and the dispersion index. (**B**) A consensus matrix of NMF clustering. (**C**) The distribution of the IDH1 mutation status across the clusters. (**D**) The distribution of the molecular subtypes across the clusters. (**E**) Kaplan–Meier survival curves for the overall survival associated with the clusters.

To determine whether cytotoxic T-lymphocyte immune-evasion-associated genes (CEAGs) exhibit significant transcriptional heterogeneity in glioma patients, we performed non-negative matrix factorization (NMF) on 525 samples from the TCGA-GBM-HG_U133A dataset using 182 CEAGs derived from Lawson et al. [19] as input features. Notably, historical datasets (e.g., those based on the 2016 WHO classification) classified all WHO grade-4 astrocytic tumors as “GBM”, regardless of their IDH status. Under the current diagnostic criteria, IDH-mutant tumors are no longer classified as GBM but as distinct astrocytoma entities. In this study, we retain the term “GBM” exclusively for IDH-wildtype tumors, in line with the 2021 WHO criteria, while acknowledging that historical data may include a minor subset of IDH-mutant cases now reclassified as astrocytoma, IDH-mutant. As shown in Figure 1A, the cophenetic coefficient and dispersion index jointly indicated optimal factorization stability at k = 2. Of the k values from 2 to 9, the results for k = 2 have the best robustness (Supplementary Figure S1). Consensus clustering based on 100 NMF iterations (Figure 1B) robustly segregated all the samples into two distinct subgroups (Consensus Cluster 1 vs. 2) according to CEAG expression patterns, demonstrating that CEAGs represent a potential transcriptional reprogramming signature for the molecular subtyping of glioma.

We further analyzed the distribution of classical prognostic molecular subtypes across CEAG-based clusters. Cluster 2 exhibited a significantly higher proportion of isocitrate dehydrogenase 1 (IDH1)-wildtype patients (98.18% vs. 85.71% in Cluster 1, Figure 1C). Notably, the mesenchymal subtype [20] predominated in Cluster 2 (52.84% vs. 6.58% in Cluster 1, Figure 1D), consistent with the established association between the mesenchymal phenotype, the IDH1-wildtype status, and an unfavorable prognosis [21,22]. A Kaplan–Meier survival analysis confirmed worse clinical outcomes in Cluster 2 patients (log-rank *p* = 0.031, Figure 1E), validating the prognostic relevance of CEAG-based classification.

**Figure 2:**
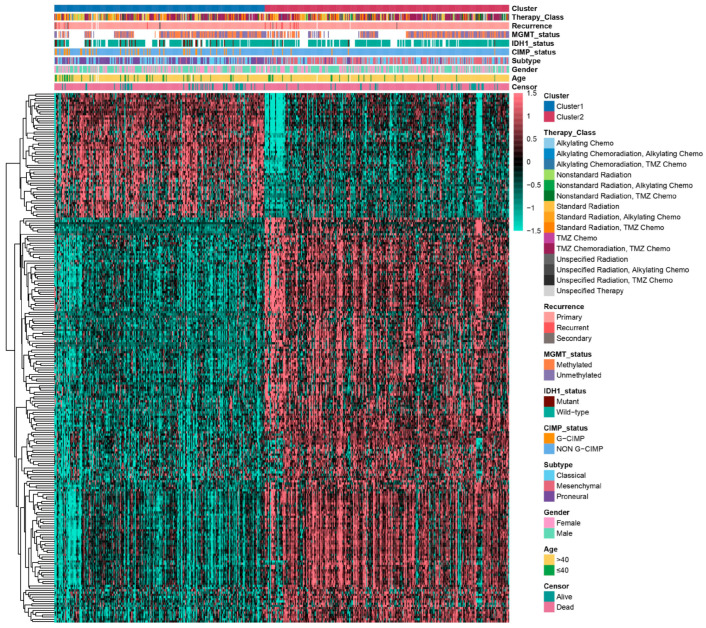
Differential gene expression distribution between glioma subtypes. Heatmap of 238 DEGs between Cluster 1 and Cluster 2, with clinicopathological annotations.

A limma differential expression analysis (|log2 fold change| > 1, adjusted *p* < 0.05) identified 238 significantly deregulated genes (DEGs) between clusters. A heatmap visualization (Figure 2) revealed distinct transcriptional landscapes and clinicopathological correlations across the CEAG subtypes. Intriguingly, the glioma-associated CpG island methylator phenotype (G-CIMP), a molecular signature linked to a favorable prognosis [23], was under-represented in Cluster 2. These findings collectively establish CEAGs as robust biomarkers for glioma stratification and preliminarily demonstrate their prognostic predictive potential.

### 2.2. Biological Function Analysis of Glioma Subtypes Mediated by Cytotoxic T-Lymphocyte Immune Evasion Dynamics

The transcriptional reprogramming driven by the differential expression of CTLE-associated genes may activate multiple signaling pathways and biological processes, thereby enabling tumor cells to evade cytotoxic T-lymphocyte-mediated killing and promote malignant progression [19]. To delineate the biological heterogeneity between CTLE-mediated glioma subtypes, we conducted multidimensional assessments encompassing molecular functions, immune infiltration, tumor stemness, and cancer-related pathways.

**Figure 3:**
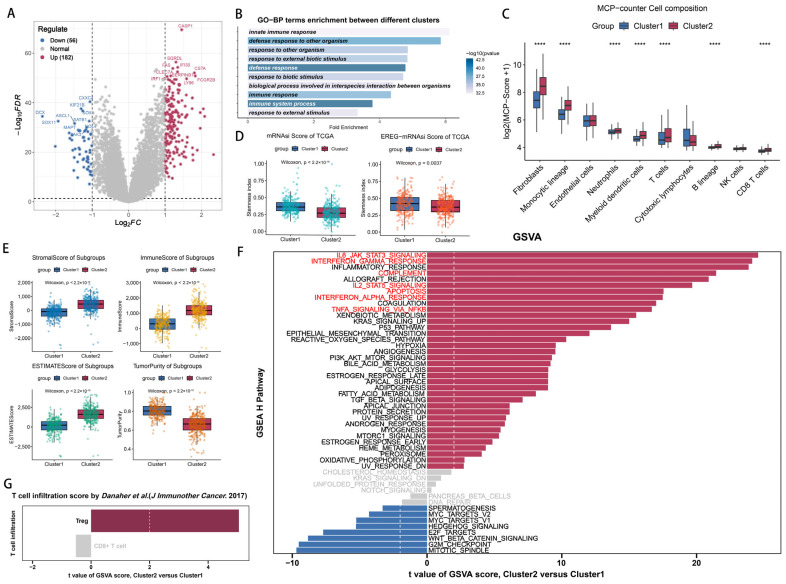
Biological function analysis of glioma subtypes mediated by cytotoxic T-lymphocyte immune evasion dynamics. (**A**) Volcano plot of DEGs. (**B**) GO-BP enrichment analysis of DEGs. (**C**) Immune cell infiltration estimated by MCP-counter. (**D**) Tumor stemness scores (mRNAsi and EREG-mRNAsi). (**E**) ESTIMATE scores for stromal, immune, and tumor purity. (**F**) GSVA of HALLMARK pathways. (**G**) GSVA of Treg and CD8+ T-cell infiltration [25]. **** *p*  <  0.0001; ns, non-significant.

A differential expression analysis revealed 182 upregulated and 56 downregulated genes (DEGs) among the subtypes (Figure 3A). A Gene Ontology-Biological Process (GO-BP) enrichment analysis demonstrated the considerable enrichment of DEGs in the innate immune response, a defense response to other species, and immune system processes (Figure 3B), corroborating the findings of Lawson et al. [19]. An MCP-counter-based immune deconvolution revealed elevated infiltration levels of T-cells, CD8+ T-cells, fibroblasts, monocytic lineage cells, neutrophils, dendritic cells, and B cells in Cluster 2 (Figure 3C), underscoring the divergent immune milieu landscapes among the subtypes.

A tumor stemness evaluation using the OCLR algorithm [24] showed reduced stemness-related scores (mRNAsi and EREG-mRNAsi) in Cluster 2 (Figure 3D). ESTIMATE-algorithm-based immune scoring further revealed elevated stromal scores, enhanced immune activity, and reduced tumor purity in Cluster 2 compared to Cluster 1 (Figure 3E). A Gene Set Variation Analysis (GSVA) of HALLMARK pathways (Figure 3F) demonstrated the significant upregulation of immune-evasion-related pathways in Cluster 2, including IL6-JAK-STAT signaling, TNFα signaling, IL2-STAT5 signaling, interferon-α/γ response, complement activation, and apoptosis (all *p* < 0.05), aligning with prior mechanistic studies [19]. To further assess the status of T-cell immune activation versus immunosuppression between clusters, we referred to the previous work of Danaher et al. [25] and assessed the level of immunosuppressed and cytotoxic T-cells in Cluster 1 and Cluster 2 by the GSVA algorithm using a predefined set of genes characterizing Tregs and activated CD8+ T-cells (Figure 3G). The results showed that the level of Treg infiltration was significantly upregulated in Cluster 2 compared to Cluster 1, but there was no significant difference in the infiltration index of CD8+ T-cells. This further suggests that even with the presence of CD8+ T-cells in Cluster 2 comparable to that in Cluster 1, its immunosuppressive microenvironment contributed to the escape of gliomas from the killing effect of CTL.

Collectively, our multidimensional characterization of the tumor microenvironment underscores distinct biological behaviors between CTLE-mediated glioma subtypes, potentially driving divergent tumor progression trajectories.

### 2.3. The Development and Verification of a Prognostic Model Associated with Cytotoxic T-Lymphocyte Immune Evasion in Glioma

**Figure 4:**
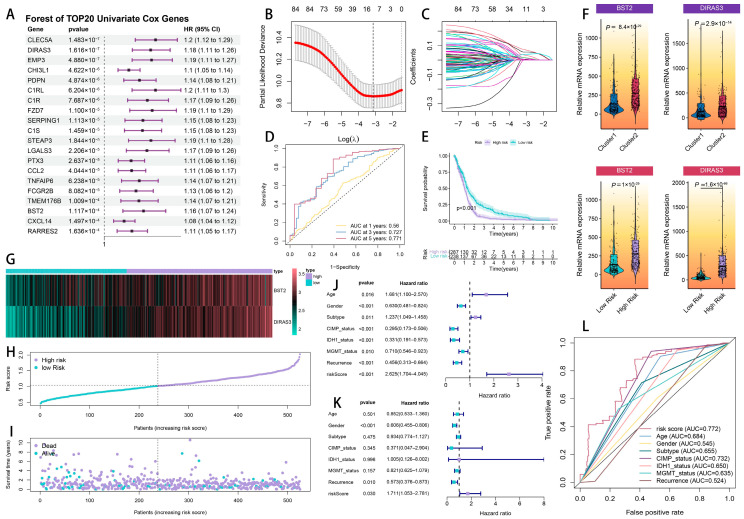
The construction of a cytotoxic T-lymphocyte immune-evasion-associated prognostic model in glioma. (**A**) A Forest plot of the top 20 univariate Cox genes. (**B**) A LASSO coefficient profile plot. (**C**) A LASSO deviance plot. (**D**) The time-dependent ROC curves for the prognostic model. (**E**) Kaplan–Meier survival curves for the high- and low-risk groups. (**F**) The expression of BST2 and DIRAS3 in the risk groups. (**G**) A heatmap of the gene expression in the risk groups. (**H**) The risk score distribution. (**I**) A survival status plot. (**J**) Univariate Cox proportional hazards regression forest plot for clinical factors and risk signature. Columns show hazard ratios (HR) with 95% confidence intervals (CI). (**K**) ASurvival distribution stratified by risk groups. Density plot shows risk score distribution with vertical dashed line separating low-risk (blue) and high-risk (red) groups. Kernel density estimation demonstratesear separation between prognostic categories. (**L**) Time-dependent receiver operating characteristic (ROC) curves at 3 years. The risk signature (red curve) demonstrates the highest predictive accuracy (AUC = 0.769) compared to individual clinical factor.

To develop a CTLE-associated prognosis model, we initially conducted a univariate Cox regression analysis on 238 DEGs within the TCGA cohort, revealing 85 genes with possible prognostic significance (Supplementary Table S1). Figure 4A presents the hazard ratios (HRs) for the top 20 DEGs, ordered by significance. Baseline patient characteristics are delineated in Table 1.

To mitigate overfitting, we applied Least Absolute Shrinkage and Selection Operator (LASSO) regression for dimensionality reduction. The coefficient profile plot (Figure 4B) illustrates the trajectory of the LASSO coefficients across varying penalty parameters (λ), with the optimal λ selected at the minimum partial-likelihood deviance (log(λ) = −3.2). This process refined the model to 11 prognostic DEGs (Figure 4C, Supplementary Table S2). A subsequent multivariate Cox regression further identified BST2 and DIRAS3 as independent risk factors (*p* < 0.05), yielding the final prognostic signature:

Risk Score (RS) = (0.124 × BST2 expression) + (0.148 × DIRAS3 expression)

All the sample RS scores in the TCGA cohort are displayed in Supplementary Table S3. The time-dependent receiver operating characteristic (ROC) analysis exhibited a strong predictive efficacy of the 2-CEAG model, with area-under-the-curve (AUC) values of 0.56, 0.727, and 0.771 for overall survival at 1, 3, and 5 years, respectively (Figure 4D). The patients that were categorized by a median RS into high- and low-risk groups demonstrated markedly different survival outcomes (log-rank *p* < 0.01, Figure 4E). Both BST2 and DIRAS3 were significantly increased in the high-risk groups across the CTLE subtypes (Wilcoxon *p* < 0.01, Figure 4F).

The correlation between RS and clinical outcomes was further illustrated by expression patterns (Figure 4G), the risk score distribution (Figure 4H), and survival status mapping (Figure 4I), indicating that an increased RS was associated with reduced survival. A multivariate Cox regression established RS as an independent prognostic factor (AUC = 0.772), outperforming classical predictors, including IDH1 mutation, MGMT methylation, G-CIMP status, and molecular subtypes.

**Figure 5:**
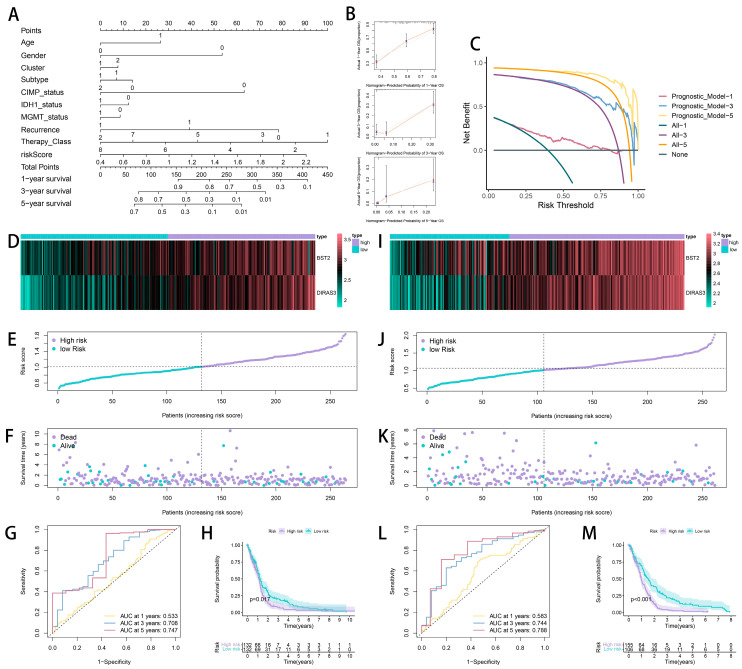
A nomogram and the validation of the prognostic model. (**A**) A nomogram integrating the risk score and clinicopathological variables. (**B**) Calibration curves for 1-, 3-, and 5-year survival. (**C**) The decision curve analysis (DCA). (**D**–**M**) The internal and external validation results.

To facilitate clinical translation, we developed a nomogram integrating the RS and clinicopathological variables (Figure 5A). Bootstrap validation (1000 resamples) confirmed the excellent calibration between the predicted and the observed 1-, 3-, and 5-year survival rates (Figure 5B). A decision curve analysis (DCA) revealed a substantial net clinical benefit when applying the nomogram for risk-guided therapeutic interventions (Figure 5C).

**Figure 6:**
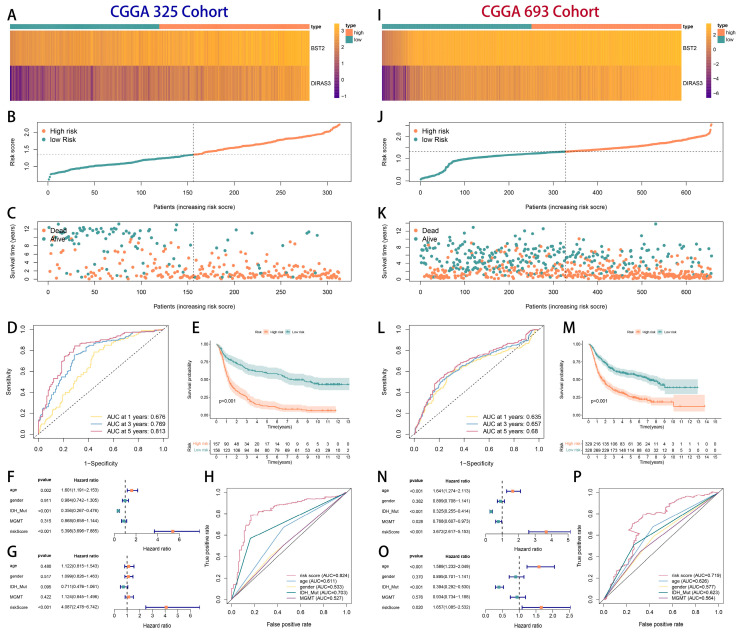
The external validation of the prognostic model. Kaplan–Meier survival curves and ROC curves in the CGGA-325 and the CGGA-693 cohorts. (**A**,**I**) Gene expression heatmaps (BST2, DIRAS3) stratified by high/low risk groups. Color gradients represent relative expression levels. (**B**,**J**) Distributions of calculated risk scores across patients. Red and blue dots/tick marks indicate high-risk and low-risk patients, respectively. Vertical dashed lines separate the groups. Survival status (dead/alive) is shown below (Panels (**C**,**K**)). (**C**,**K**) Kaplan-Meier survival curves demonstrating significantly shorter overall survival for high-risk patients compared to low-risk patients in both cohorts (Log-rank test *p* < 0.001). (**D**,**L**) Time-dependent receiver operating characteristic (ROC) curves at 1, 3, and 5 years. Area Under the Curve (AUC) values quantify the predictive accuracy of the risk score (e.g., 3-year AUC = 0.769 in CGGA 325; 3-year AUC = 0.657 in CGGA 693). (**E**,**M**) Survival probability stratified by risk group across increasing risk scores. (**F**,**G**,**N**,**O**) Forest plots of univariate (**F**,**N**) and multivariate (**G**,**O**) Cox proportional hazards regression analyses. Hazard Ratios (HR) with 95% confidence intervals demonstrate the risk score, age, IDH mutation status, and MGMT methylation status (*p* < 0.05) are independent prognostic factors for overall survival. (*HR* > 1 indicates increased risk). (**H**,**P**) Precision-Recall (PR) curves evaluating model discrimination performance (AUC values shown).

Internal validation via random cohort splitting (TCGA 50% subsampling) demonstrated consistent model performance across the subgroups (Figure 5D–M). External validation using the CGGA-325 (n = 325) and the CGGA-693 (n = 693) cohorts further corroborated the model’s generalizability (Figure 6), with concordant survival stratification and AUC metrics.

### 2.4. Tumor Heterogeneity and Microenvironment Landscape of CTLE-Associated Prognostic Models in Glioma

**Figure 7:**
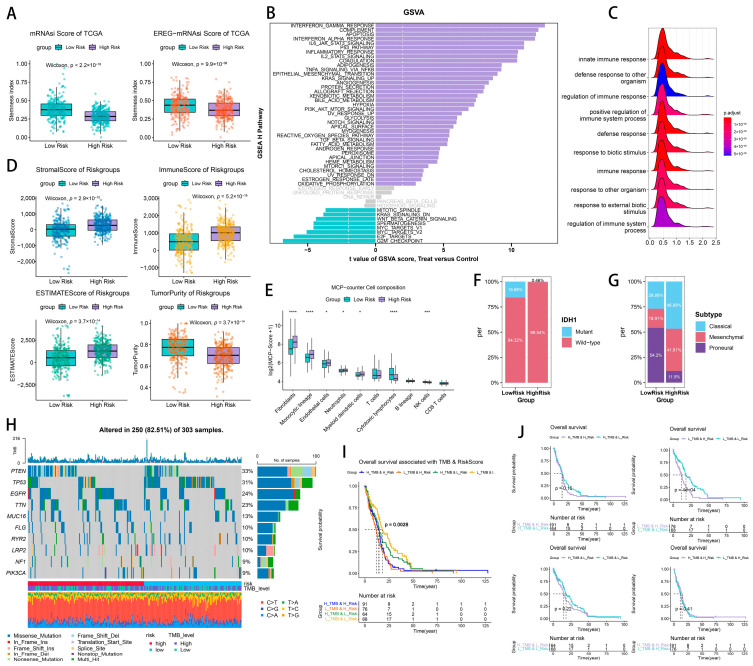
The tumor heterogeneity and microenvironment landscape of the prognostic model. (**A**) Stemness scores in risk groups. (**B**) ESTIMATE scores in risk groups. (**C**) The GSVA of HALLMARK pathways in risk groups. (**D**) A GO enrichment analysis. (**E**) MCP-counter immune infiltration. (**F**) The somatic mutation landscape. (**G**) The molecular subtype distribution. (**H**) A TMB waterfall plot. (**I**,**J**) Kaplan–Meier curves stratified by TMB and risk scores. * *p*  <  0.05; *** *p*  <  0.001; **** *p*  <  0.0001; ns, non-significant.

The mRNAsi score and EREG-mRNAsi score derived from the TCGA data were evaluated for the high- and low-risk groups, revealing a downregulation of the stemness score in the patients classified as high-risk (Figure 7A). The immune infiltration score derived from the ESTIMATE algorithm indicated that the high-risk group exhibited elevated stromal cell scores, enhanced immune response levels, and diminished tumor purity relative to the low-risk group. The gene set variant analysis (GSVA) of the samples from different subgroups using the HALLMARK pathway set from the MSigDB database, which is highly correlated with tumors, showed that pathways associated with immune escape, such as the interferon gamma response, complement response, apoptosis, interferon alpha response, IL6-JAK-STAT3 pathway, and P53, were significantly upregulated in the patients with high risk scores. The gene set enrichment analysis (GSEA) of the GO-BP terms (Figure 7C) indicated that the innate immune response, defense response to other organisms, regulation of immune response, and positive regulation of immune system processes play a pivotal role in innate immune defense and immunological regulation.

The MCPcounter analysis (Figure 7E) indicated that the infiltration of fibroblasts, monocyte lineage cells, endothelial cells, neutrophils, and myeloid dendritic cells exhibited a positive correlation with RS, with fibroblasts secreting ECM, which facilitates tumor sclerosis, invasion, and metastasis, typically indicating a poor prognosis. A significant invasion of monocyte lineage and endothelial cells typically contributes to immune evasion by the tumor. Elevated neutrophil infiltration is linked to an inflammatory microenvironment and may facilitate the epithelial–mesenchymal transition (EMT). In conclusion, substantial changes in the tumor immune microenvironment and the types of immune cell infiltration between the high-risk and low-risk patient groups are correlated with RS and may significantly influence the patients’ prognoses.

According to the somatic mutation data (Figure 7F), the molecular classification of the patients in the high-risk category resembled the IDH1-wildtype. Figure 7G illustrates that the subtype classification in the high-risk group had an approximately equal distribution between the classic and mesenchymal types, with the classic type comprising a slightly larger proportion. The low-risk group exhibited the largest proportion of subtyping as the proneural type. Figure 7H illustrates that in the TMB waterfall plots of the various risk subgroups within the TCGA HGG patient cohort, the ten genes with the highest mutation frequencies were PTEN (33%), TP53 (31%), EGFR (24%), TTN (23%), MUC15 (13%), FLG (10%), RYR2 (10%), LRP2 (10%), NF1 (9%), and PIK3CA (9%). In the high-risk subgroup, the TMB score was elevated, with PTEN exhibiting the highest mutation frequency, demonstrating significant alterations between the high- and low-risk groups, markedly exceeding those of the low-risk group. High-frequency mutations in PTEN, functioning as an oncogene, typically indicate loss-of-function mutations. TP53, exhibiting the second-greatest mutation frequency, demonstrated significant alterations between the high- and low-risk groups, with a markedly higher prevalence in the low-risk group compared to the high-risk group. Frequent mutations in TP53 forecasted a decline in genomic stability and facilitated tumor growth.

Therefore, by doing K–M survival curves for the different risk subgroups, combined with TMB-scoring the subgroups of glioma patients in the TCGA (Figure 7I,J), we found that the low-risk subgroups have a prognostic potency for glioma patients, which is reflected more significantly in the low-TMB subgroups (Figure 7J, top, *p* < 0.01), and similarly, a low TMB has prognostic potency for glioma patients, which is reflected in low-risk subgroups more significantly (Figure 7J, low, *p* < 0.05).

### 2.5. Immune Checkpoint Signatures of CTLE-Associated Prognostic Models in Glioma

**Figure 8:**
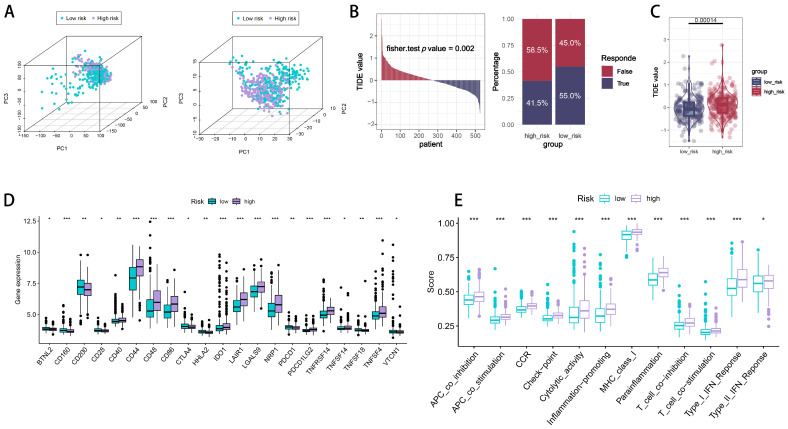
Immune checkpoint signatures of the prognostic model. (**A**) The principal component analysis (PCA) of the gene expression of the TCGA cohort (left) and the NMF subgroup of CTLE (right). Differences in ssGSEA immune infiltration analysis and immune-checkpoint-associated gene expression between the high- and low-risk subgroups. (**B**) The distribution of TIDE immune escape scores with the corresponding levels of immunotherapy in the high- and low-risk groups. (**C**) A violin plot of TIDE scores in different risk groups. (**D**) The differential expression of immune-checkpoint-associated genes. (**E**) An immune-related functional analysis. * *p*  <  0.05; ** *p*  <  0.01; *** *p*  <  0.001; ns, non-significant.

To reconfirm the robustness of the CTLE-associated genes in defining the differential transcriptional patterns of all the glioma samples, we performed principal component three-dimensional analyses for all the samples. As shown in Figure 8A, all the genes from the TCGA cohort (Figure 8A, left) and 286 differential genes from the NMF subgroup of the CTLE (Figure 8A, right) were plotted to illustrate the distribution of gene expression across various dimensions. The findings indicated a strong correlation between the risk subgroups and the PCA analysis results in both the TCGA complete gene set and the 286 differential genes. To further assess the level of immune escape in different risk groups, we performed the Tumor Immune Dysfunction and Exclusion (TIDE) algorithm on all the glioma samples to predict the patient response to immune checkpoint inhibitors. The results showed significant differences in the TIDE scores between the different risk groups, with the patients in the high-risk group having a poorer immune response and tending to have an immune escape tumor phenotype (Figure 8B). Subsequent statistical tests of the TIDE scores of the two groups showed that the patients in the high-risk group had significantly higher TIDE scores (*p* < 0.01), suggesting a higher likelihood of immune escape from their tumors and potentially less benefit from immune checkpoint inhibitors. The stratified analysis of immune-checkpoint-gene-related genes (Figure 8D) demonstrated that the expression levels of immune checkpoint genes CD44, CD48, CD86, LAIR1, LGALS9, NRP1, TNFRSF14, and TNFSF4 were significantly elevated in the high-risk group compared to the low-risk group (*p* < 0.05), indicating that the patients in the high-risk group may exhibit a more favorable response and prognosis to inhibitors targeting these immune checkpoints. Consequently, the ssGSEA package was utilized to evaluate the relationship between risk ratings and immune cells, along with their roles.

Immune function scores (Figure 8E), including APC co-inhibition, APC co-stimulation, CCR (C-C chemokine receptor), immune checkpoints, cytolytic activity, inflammation promotion, MHC Class I, parainflammation, T-cell co-inhibition, T-cell co-stimulation, and type I and type II interferon responses, were elevated in the high-risk group relative to the low-risk group. This indicates that, unlike the low-risk-scoring population, patients in the high-risk group exhibited sensitivity to immune checkpoint inhibitors due to an imbalance in antigen-presenting cell co-suppression/co-stimulation, T-cell co-suppression/co-stimulation, and the elevated expression of immune checkpoints, resulting in a predominance of immune escape and an inhibitory microenvironment. Chronic inflammation and a pro-tumor immune response are evidenced by elevated inflammation-promoting and parainflammatory signals, along with increased CCR expression. The tumor microenvironment in the high-risk cohort may be “immune-activated yet functionally suppressed”, indicating that integrated immunotherapy and anti-inflammatory strategies could yield greater efficacy.

Additionally, we explored drug sensitivity predictions in the high-risk group, identifying six drugs—KU-55933, NU7441, PD0325901, AZD6482, AZD8186, and ENTOSPLETINIB—with lower IC50 values compared to the low-risk group (Supplementary Figure S2A). These drugs have not been reported or utilized in high-grade glioma (HGG) treatment, suggesting potential future clinical applications. Furthermore, three drugs—AZD8055, SELUMETINIB, and TRAMETINIB—exhibited lower IC50 values in the high-risk group than in the low-risk group (Supplementary Figure S2B), consistent with prior HGG studies. While these findings lack experimental validation, they may offer valuable insights for investigating treatment-resistant phenotypes in glioma.

### 2.6. BST2/DIRAS3 Knockdown Suppresses Glioma Invasion and Migration

**Figure 9:**
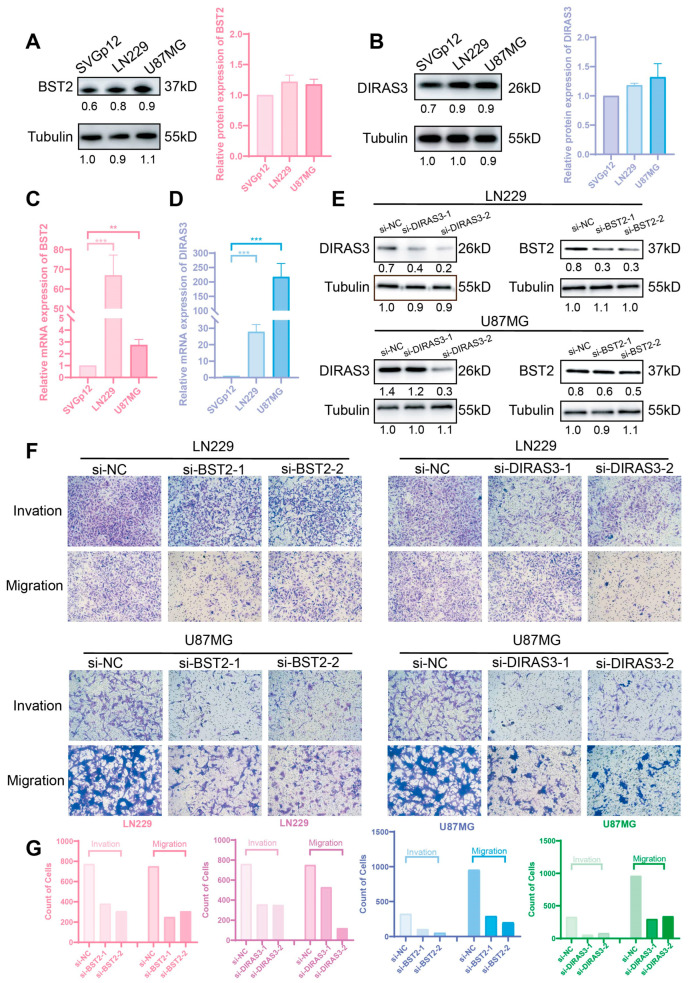
The expression of BST2 and DIRAS3 in normal and GBM cells. The effects of the inhibition of BST2 and DIRAS3 on GBM cells. (**A**,**B**) The protein expression of BST2 and DIRAS3 in normal and GBM cells. (**C**,**D**) The mRNA expression of BST2 and DIRAS3 in normal and GBM cells. (**E**) The effect of the inhibition of BST2 and DIRAS3 in the GBM cell line. (**F**) The inhibition of BST2 and DIRAS3 decreased GBM cell invasion and migration ability in the invasion and migration assay. The quantification analysis of invasion cells and migration cells (**G**) in different groups. ** *p*  <  0.01; *** *p*  <  0.001; ns, non-significant.

This study expands upon our previous findings about the roles of BST2 and DIRAS3 in the evasion of the glioma immune milieu by examining their direct regulatory effects on tumor cell invasion and migratory capabilities. Preliminary comparative expression investigation by qRT-PCR and Western blot demonstrated markedly increased expression levels of both genes in glioma cell lines (LN229 and U87MG) relative to normal human astrocytes (svgP12) (Figure 9A–D). To clarify their functional significance, the siRNA-mediated knockdown of BST2/Diras3 was conducted. Subsequent transwell experiments revealed that the genetic silencing of these two genes markedly diminished the invasive and migratory activities of both LN229 and U87MG cells (Figure 9E–G).

## 3. Discussion

Gliomas represent the most prevalent primary malignant brain tumors worldwide, with HGG constituting their most aggressive subtype [3]. Distinct from solid tumors in other organs, HGGs exhibit unique pathological characteristics that confer therapeutic challenges [3,6,7,26]. Prior to tumor invasion, the blood–brain barrier (BBB) functions as a semipermeable membrane that substantially restricts immune cell trafficking into the brain parenchyma [27]. Additionally, the glioma microenvironment is characterized by predominant immunosuppressive cellular populations—including tumor-associated macrophages/microglia (TAM/MG), myeloid-derived suppressor cells (MDSCs), and regulatory T-cells (Tregs)—which synergistically suppress antitumor immunity [9,28,29,30,31,32,33]. These pathophysiological features collectively underlie the marked therapeutic resistance and dismal prognosis associated with HGGs. Consequently, elucidating the core regulatory genes within the HGG immune microenvironment will profoundly impact our understanding of tumor immune evasion and lay the groundwork for developing novel, targeted therapies.

Through a functional enrichment analysis, this study identified BST2 and DIRAS3 as pivotal regulatory genes associated with CD8+ T-cell-mediated immune evasion and clinical prognoses in HGGs. Mechanistic investigations suggest these genes may drive HGG immune escape by mediating immunosuppressive signaling within the tumor microenvironment. Functional validation experiments demonstrated that the knockdown of BST2 or DIRAS3 expression in glioma cells significantly suppresses their in vitro migration and invasion capabilities, confirming their critical regulatory roles in malignant phenotypic progression. Certainly, it must be confirmed whether this is as definitive in mice tumor models as it is in vitro, as it was only experimentally validated in glioblastoma cell lines. The migratory and invasive potential of BST2/DIRAS3 in glioblastoma has only been validated in vitro by our current studies; pertinent marker assays that target the tumor microenvironment are required.

BST2, serving as a functional ligand for the ILT7 receptor, modulates tumor immune responses through a dual mechanism: firstly, by binding to the ILT7 receptor, it inhibits the secretion of type I interferon (IFN) mediated by TLR9/TLR7 in plasmacytoid dendritic cells (pDCs), thereby weakening their antiviral and antitumor activity; secondly, it synergistically enhances the inhibition of pDC activation through interaction with the transmembrane adhesion receptor CD44 [34]. This immunomodulatory function manifests as a pro-cancer effect in various malignant tumors: in ER-negative breast cancer, the high expression of BST2 drives tumor cell migration and invasion [35]; in CRC, it promotes the formation of an immunosuppressive microenvironment by inducing the M2 polarization of tumor-associated macrophages (TAMs) [15]; in pancreatic cancer, BST2 binds to PD-1, activating the PI3K/AKT pathway, leading to the functional exhaustion and apoptosis of CD8+ T-cells [36]. Additionally, studies on oral squamous cell carcinoma (OSCC) have shown that BST2 not only directly promotes the malignant phenotype of tumor cells but also suggests its role in remodeling the tumor immune microenvironment, as indicated by the enrichment characteristics of its immune-related pathways [37].

In addition to BST2, another key regulatory gene identified in our study is DIRAS3, which exhibits multifaceted functional complexity in oncogenesis. DIRAS3, an imprinted tumor suppressor gene intricately associated with autophagy, activates this process through mechanisms including the inhibition of the PI3K/AKT/mTOR signaling axis, the promotion of the Beclin1 complex assembly, and the maintenance of FOXO3a nuclear localization [17]. While autophagy exerts tumor-suppressive effects by inducing cell death under sustained stress conditions, emerging evidence highlights its context-dependent pro-survival and immunomodulatory roles during tumor progression [38,39]. Studies demonstrate that in ovarian cancer microenvironments [40], DIRAS3-dependent autophagy sustains dormant cancer cell viability under nutrient deprivation, suggesting its involvement in therapy resistance through metabolic adaptation. In CRC, autophagy initially maintains genomic stability via mutagen clearance during early stages, but transitions to promoting metastasis in advanced disease by alleviating metabolic stress and driving epithelial–mesenchymal transition (EMT) [41]. Crucially, autophagy that can be regulated by DIRAS3 is mechanistically shown to contribute to tumor immune evasion via the lysosomal degradation of MHC class I molecules, thereby reducing the antigen presentation efficiency and impairing CD8+ cytotoxic T-lymphocyte (CTL)-mediated tumor elimination [42,43].

Integrating prior research with our current findings, we establish BST2 and DIRAS3 as key regulatory genes governing T-cell-mediated immune evasion in glioma. However, this study is constrained by insufficient in vivo data to delineate their precise roles in the dynamic remodeling of the glioma immune microenvironment; we also cannot fully elucidate their mechanistic regulation of tumor migration/invasion. Based on published research and our findings, we hypothesize a dual-mode mechanism: (1) BST2/DIRAS3 may activate pro-invasive signaling pathways intrinsically within glioma cells; (2) their functional synergy within the tumor microenvironment (TME) potentially establishes systemic immune evasion, particularly against CD8+ T-cell-mediated antitumor immunity. Future research should focus on validating these hypotheses and exploring the therapeutic potential of targeting BST2 and DIRAS3 in glioma treatment.

## 4. Materials and Methods

### 4.1. Data Sources

This study utilized transcriptomic data from The Cancer Genome Atlas (TCGA) and the Chinese Glioma Genome Atlas (CGGA). Specifically, the TCGA-GBM-HG_U133A dataset, comprising 525 glioma samples, was employed for the initial clustering and the gene expression analysis. Two external validation cohorts from the CGGA were included: CGGA-325 (n = 325) and CGGA-693 (n = 693). The analysis focused on 182 cytotoxic T-lymphocyte immune-evasion-associated genes (CEAGs) derived from Lawson et al. [19]. The HALLMARK pathway set from the Molecular Signatures Database (MSigDB) was used for the pathway analysis. Additionally, the TCGA provided somatic mutation data and clinical outcomes (e.g., survival data) to assess tumor heterogeneity and prognostic factors.

### 4.2. Data Preprocessing

Transcriptomic data from the TCGA-GBM-HG_U133A dataset underwent standard bioinformatics preprocessing, including normalization and batch effect correction, to ensure the data quality. The 182 CEAGs were selected as key features for subsequent analyses. Clinical data, such as survival time and molecular subtype, were integrated with the transcriptomic data to ensure analytical consistency. The Least Absolute Shrinkage and Selection Operator (LASSO) regression was utilized in the construction of prognostic models to reduce dimensionality and address overfitting concerns.

### 4.3. Non-Negative Matrix Factorization (NMF)

Non-negative matrix factorization (NMF) was performed to identify transcriptional heterogeneity among the 525 TCGA samples based on the 182 CEAGs. The analysis utilized the NMF package (version 0.28) in R. The optimal number of clusters, k = 2, was established through the application of the cophenetic correlation coefficient and dispersion index. Consensus clustering, with 100 iterations, segregated the samples into two distinct subgroups: Cluster 1 and Cluster 2.

### 4.4. Differential Expression Analysis

The limma package (version 3.54.0) was utilized to perform a differential expression analysis between the two NMF clusters. Genes exhibiting |log2 fold change| > 1 and an adjusted *p*-value < 0.05 were classified as differentially expressed genes (DEGs). A total of 238 DEGs were identified and visualized through a volcano plot to emphasize expression differences. These DEGs were further used for a functional analysis and a prognostic evaluation.

### 4.5. Biological Function and Pathway Analysis

An enrichment analysis of the Gene Ontology-Biological Process (GO-BP) was conducted on the DEGs utilizing the clusterProfiler package (version 4.6.2) to identify enriched biological processes, including innate immune response and immune system processes. A gene set enrichment analysis (GSEA) for GO-BP terms was performed utilizing the gseGO function from the clusterProfiler package (version 4.6.2). In GSEA, the genes were ranked according to their log2 fold change values derived from the differential expression analysis. This analysis evaluated the enrichment of GO-BP terms across the ranked gene list, providing insights into biological processes significantly associated with gene expression changes between the subtypes. A Gene Set Variation Analysis (GSVA) was performed utilizing the GSVA package (version 1.46.0) alongside the MSigDB HALLMARK pathway set and Tregs and activated CD8+ T-cells gene sets to evaluate pathway activity and immune cell infiltration. The results revealed the significant upregulation of immune-evasion-related pathways (e.g., IL6-JAK-STAT, TNFα signaling) in Cluster 2.

### 4.6. Immune Infiltration Analysis

Immune cell infiltration was assessed using the Microenvironment Cell Populations-counter (MCP-counter) method through the MCPcounter package (version 1.2.0, obtained from GitHub) and the ESTIMATE algorithm via the estimate package (version 1.0.13). The MCP-counter method quantified the abundance of various immune cell types, including T-cells, CD8+ T-cells, and fibroblasts, while ESTIMATE generated stromal and immune scores, along with tumor purity estimates. Cluster 2 demonstrated elevated immune and stromal scores alongside reduced tumor purity.

### 4.7. Prognostic Model Construction

The prognostic model was developed using the subsequent steps:

1. Univariate Cox Regression: Applied to the 238 DEGs using the survival package (version 3.5-5), identifying 85 genes with a potential prognostic value.
2. LASSO Regression: Conducted using the glmnet package (version 4.1-8) to reduce variables to 11 prognostic DEGs, with the optimal penalty parameter (λ) selected at log(λ) = −3.2.
3. Multivariate Cox Regression: Identified BST2 and DIRAS3 as independent risk factors, constructing a risk score (RS) formula: RS = (0.124 × BST2 expression) + (0.148 × DIRAS3 expression).

The evaluation of model performance was conducted through time-dependent receiver operating characteristic (ROC) curves utilizing the timeROC package (version 0.4). The area-under-the-curve (AUC) values obtained were 0.56, 0.727, and 0.771 for overall survival at 1, 3, and 5 years, respectively. Survival outcomes were compared between the high-risk and low-risk groups using a Kaplan–Meier survival analysis. Internal validation utilized a 50% subsampling of the TCGA dataset, while external validation was carried out with the CGGA-325 and CGGA-693 cohorts. A nomogram that combines the risk score with clinicopathological variables was developed using the rms package (version 8.0-0). It was validated through 1000 bootstrap resamples for calibration curves and evaluated for clinical utility using a decision curve analysis (DCA) with the rmda package (version 1.5).

### 4.8. Tumor Heterogeneity and Microenvironment Analysis

Tumor stemness was evaluated using the One-Class Logistic Regression (OCLR) algorithm to compute mRNAsi and EREG-mRNAsi scores, implemented via custom scripts based on Malta et al. [24]. Immune infiltration was further characterized using the MCP-counter and ESTIMATE algorithms. A somatic mutation analysis was performed using the maftools package (version 2.14.0) to examine mutation frequencies (e.g., PTEN, TP53) and their associations with the risk groups, visualized using tumor mutation burden (TMB) waterfall plots and Kaplan–Meier survival curves.

### 4.9. Principal Component Analysis

A principal component analysis (PCA) was carried out employing the factoextra package (version 1.0.7) to illustrate the gene expression distributions among the risk subgroups.

### 4.10. Drug Sensitivity and Immune Checkpoint Analysis

Expression differences in immune checkpoint genes (e.g., CD44, CD48) and their potential responsiveness to immune checkpoint inhibitors were analyzed. Single-sample gene set enrichment analysis (ssGSEA) was executed utilizing the GSVA package to associate risk scores with immune functions (e.g., antigen-presenting cell co-inhibition, T-cell co-stimulation). TIDE (Tumor Immune Dysfunction and Exclusion) is an algorithm for predicting the response to immune checkpoint inhibitors (e.g., PD-1/PD-L1 inhibitors) in cancer patients by examining the correlation between the expression of each gene in a tumor modeling cohort, the level of effector-toxicity T-lymphocyte (CTL) infiltration, and the impact on the survival situation, thereby constructing algorithms to characterize T-cell dysfunction in other tumor cohorts. Through the portal (http://tide.dfci.harvard.edu/ (accessed on 19 May 2024)), we submitted the preprocessed expression matrices as requested and visualized them using the R package ggplot2 (version 3.5.2). Drug sensitivity was predicted using the oncoPredict package (version 0.2, sourced from GitHub, San Francisco, CA, USA) through the Genomics of Drug Sensitivity in Cancer (GDSC) V2 training dataset to compare IC50 values between the high- and low-risk groups to identify potential therapeutic agents.

### 4.11. Statistical Analysis

Statistical analyses were performed using R 4.3.1 (Bioconductor 3.17). Survival outcomes were compared via log-rank tests, and continuous variables were analyzed using Wilcoxon rank-sum tests. A *p*-value < 0.05 (two-sided) was considered statistically significant.

### 4.12. Software and Packages

The following R packages were used in this study, with versions compatible with R 4.3.1 and Bioconductor 3.17:

1. NMF (version 0.28): For NMF clustering.
2. limma (version 3.54.0): For differential expression analysis.
3. survival (version 3.5-5): For survival analysis and Cox regression.
4. glmnet (version 4.1-8): For LASSO regression.
5. timeROC (version 0.4): For time-dependent ROC analysis.
6. clusterProfiler (version 4.6.2): For GO enrichment analysis.
7. GSVA (version 1.46.0): For GSVA and ssGSEA.
8. MCPcounter (version 1.2.0, GitHub): For immune cell infiltration estimation.
9. estimate (version 1.0.13): For stromal, immune, and tumor purity scores.
10. maftools (version 2.14.0): For somatic mutation analysis.
11. oncoPredict (version 0.2, GitHub): For drug sensitivity prediction.
12. ComplexHeatmap (version 2.14.0): For heatmap visualization.
13. pheatmap (version 1.0.12): For heatmap visualization.
14. rms (version 8.0-0): For nomogram construction.
15. rmda (version 1.5): For decision curve analysis.
16. factoextra (version 1.0.7): For PCA visualization.

These packages were installed and utilized within the R environment version 4.3.1.

### 4.13. Ethical Considerations

As this study utilized publicly available datasets from the TCGA and the CGGA, no additional ethical approvals were required. All the data were accessed and used in compliance with the respective data providers’ policies.

### 4.14. Cell Culture and Treatment

The human brain astrocyte normal cell line svgP12 was obtained from Cellverse (cat# iCell-h555, Shanghai, China). The human glioma cell lines U87MG (cat# CM-H874) and LN229 (cat# CM-H602) were obtained from GAINING BIOLOGICAL, Shanghai, China. These cells were cultured in Dulbecco modified Eagle medium (DMEM) (cat# C11995500BT, Gibco, Thermo Fisher Scientific, Waltham, MA, USA) containing 10% Fetal Bovine Serum Standard (FBS) (cat# FBS-S500, NewZerum, Christchurch, New Zealand).

### 4.15. siRNAs and Transfection

ShengGong (Shanghai, China) synthesized the siRNAs for human BST2 and DIRASF3. The cells were plated at a density of 1.5 × 10^5^ per well of a 6-well plate 18 h before transfection. For transient transfections, KeygenMax 3000 (cat# KGA9705-1.5, KeyGen BioTech, Nanjing, China) and opti-MEM (cat# 31985070, Gibco, Thermo Fisher Scientific, Waltham, MA, USA) were used per the protocols.

The following oligonucleotide sequences of siRNAs were used: negative control siRNA, 5′-UUCUCCGAACGUGUCACGUdTdT-3′ (forward), si-BST2#1, 5′-GGAGAGAUCACUACAUUAAdTdT-3′ (forward), si-BST2#2, 5′-GCGUGAGAAUCGCGGACAAdTdT-3′ (forward), si-DIRASF3#1, 5′-ACAGGAAGAUCAGAGAUUAdTdT-3′ (forward), si-DIRASF3#2, 5′-CCUGUGCGAUGGAGUGGAAdTdT-3′ (forward).

### 4.16. RNA Extraction and qRT-PCR

Total RNA extraction from cell lines was performed using the Cell Total RNA Rapid Extraction Kit (cat# 400-100, GOONIEBIO, Guangzhou, China) according to the established protocols. The reverse transcription of 1 µg of total RNA into cDNA was conducted utilizing the PrimeScript RT reagent Kit with gDNA Eraser (cat# RR047A, Takara, Otsu, Japan). Real-time PCR reactions were conducted utilizing the Thermo ABI QuantStudio6 FLEX system from Thermo, USA. The internal reference GAPDH was utilized to normalize the RT-qPCR results, with the relative expression calculated using the 2^(−ΔΔCT)^ method.

The primer pairs utilized were as follows: Human BST2, 5′-TCTCCTGCAACAAGAGCTGACC-3′ (forward) and 5′-TCTCTGCATCCAGGGAAGCCAT-3′ (reverse); human DIRASF3, 5′-CACATCACCGACAGCAAGAGTG-3′ (forward) and 5′-CAGGGTTTCCTTCTTGGTGACTG-3′ (reverse).

### 4.17. Western Blotting (WB) and Antibodies

The cells were lysed in an RIPA buffer (cat# P0013B, Beyotime, Shanghai, China) supplemented with protease inhibitors, maintained on ice. The lysates were combined with a loading buffer (cat# CW0027S, CWBIO, Shanghai, China) and denatured at 100 °C for 3 min. The products were subsequently subjected to Sodium dodecylsulfate-polyacrylamide gel electrophoresis (SDS-PAGE) followed by transfer to polyvinylidene difluoride (PVDF) membranes (Millipore, Billerica, MA, USA). The membranes underwent incubation with primary antibodies—α-Tubulin (Proteintech (Rosemont, IL, USA), 1:5000), BST2 (ZENBIO (Durham, NC, USA), 1:5000), and DIRASF3 (Abmart (Berkeley Heights, NJ, USA), 1:5000)—followed by the application of a secondary antibody conjugated to horseradish peroxidase (Beyotime, Shanghai, China). Immunoreactive bands were visualized with the Amersham Imager 680 following the application of a luminol-based chemiluminescent substrate (ECL; MeilunBio (Dalian, China)). The immunoblotting results were analyzed with Image J software.

### 4.18. Transwell Assay

First, 1 × 10^5^ LN229 and U87MG cells were resuspended in a serum-free medium and seeded onto the upper side of filters with an 8 μm pore size (cat#3422, Corning, Corning, NY, USA). The lower chamber was supplemented with 10% FBS medium and incubated at 37 °C for 24 h. Subsequently, the cells were fixed in 100% methanol at 4 °C for 10 min, and the upper side of the insert was meticulously cleaned with cotton swabs. The insert’s lower section was stained using a 0.5% solution of crystal violet. In the invasion assay, Matrigel (Corning Matrigel Basement Membrane Matrix, LDEV-free) was diluted 1:9 with an ice-cold, serum-free medium and polymerized in transwell inserts at 37 °C for a minimum of 1 h. Then, 3 × 10^5^ LN229 cells and U87MG were directly seeded onto the matrigel in a serum-free medium. Transwell inserts were subsequently placed in a medium containing 10% FBS, and the cells were permitted to invade at 37 °C for a duration of 24 h. The invading cells were fixed and processed according to the procedures outlined in the transwell migration assay section. The images were captured using an inverted microscope (Olympus IX73, Shinjuku, Japan).

## 5. Conclusions

This study systematically unveils the dual mechanistic roles of BST2 and DIRAS3 in gliomagenesis and progression: (1) As independent prognostic factors, their aberrant overexpression significantly correlates with CD8+ T-cell-mediated immune evasion and poor clinical outcomes in glioma patients. (2) They cooperatively drive tumor invasion and metastasis by remodeling the immunosuppressive microenvironment through the M2 polarization of TAMs, the functional impairment of pDCs, and the exhaustion of CTLs.

Given their evolutionarily conserved immunoregulatory functions across solid tumors, BST2 and DIRAS3 emerge as novel biomarkers for glioma immunotherapy. The dual-target inhibition strategy against this axis provides a conceptual framework to overcome current limitations in immune checkpoint inhibitor resistance, potentially redefining therapeutic paradigms for immunologically “cold” gliomas.

## Figures and Tables

**Table 1:** The characteristics of patients from the CGGA and the TCGA.

Patient Characteristics					
Characteristic	CGGA_325, *N* = 325 ^1^	CGGA_693, *N* = 693 ^1^	TCGA-Test, *N* = 261 ^1^	TCGA-Train, *N* = 264 ^1^	*p*-Value ^2^
Survival					
Mean (SD)	3.98 (4.03)	3.29 (2.74)	16.78 (17.00)	16.60 (18.50)	
Median (IQR)	1.93 (0.79, 7.04)	2.37 (0.95, 5.29)	12.20 (5.50, 20.70)	12.20 (6.00, 19.35)	
Range	0.05, 13.18	0.07, 13.78	0.10, 94.80	0.10, 127.60	
Missing	12	36	0	0	
Status	220 (70%)	397 (60%)	227 (87%)	220 (83%)	<0.001
Missing	9	30	0	0	
Patient age					
Mean (SD)	42.94 (11.95)	43.28 (12.39)	57.36 (14.34)	59.00 (14.53)	
Median (IQR)	42.00 (36.00, 51.00)	43.00 (34.00, 51.00)	58.30 (49.13, 67.98)	60.30 (50.30, 69.40)	
Range	8.00, 79.00	11.00, 76.00	10.90, 86.60	14.50, 89.30	
Missing	0	1	3	3	
Gender					0.411
Female	122 (38%)	295 (43%)	103 (40%)	100 (38%)	
Male	203 (62%)	398 (57%)	154 (60%)	160 (62%)	
Missing	0	0	4	4	
IDH1_status					<0.001
Mutant	175 (54%)	356 (55%)	16 (8.4%)	14 (6.6%)	
Wildtype	149 (46%)	286 (45%)	174 (92%)	198 (93%)	
Missing	1	51	71	52	
MGMT_status					0.031
Methylated	157 (51%)	315 (58%)	90 (51%)	80 (47%)	
Unmethylated	149 (49%)	227 (42%)	85 (49%)	92 (53%)	
Missing	19	151	86	92	
Recurrence					<0.001
Primary	229 (71%)	422 (61%)	248 (96%)	249 (95%)	
Recurrent	59 (18%)	271 (39%)	7 (2.7%)	9 (3.4%)	
Secondary	33 (10%)	0 (0%)	4 (1.5%)	3 (1.1%)	
Missing	4	0	2	3	

^1^ n (%); ^2^ Pearson’s Chi-squared test.

## Data Availability

All the transcriptome data generated or analyzed during the present study was downloaded from the TCGA (https://portal.gdc.cancer.gov, accessed on 10 September 2024) and the CGGA (http://www.cgga.org.cn/, accessed on 10 September 2024) databases, which are available freely with open access.

## Supplementary Materials

The following supporting information can be downloaded at https://www.mdpi.com/article/10.3390/ijms26136205/s1.

## References

[1] Horbinski C., Berger T., Packer R.J., Wen P.Y. (2022). Clinical implications of the 2021 edition of the WHO classification of central nervous system tumours. Nat. Rev. Neurol..

[2] Schaff L.R., Mellinghoff I.K. (2023). Glioblastoma and Other Primary Brain Malignancies in Adults: A Review. JAMA.

[3] Weller M., Wen P.Y., Chang S.M., Dirven L., Lim M., Monje M., Reifenberger G. (2024). Glioma. Nat. Rev. Dis. Primers.

[4] Wen P.Y., Weller M., Lee E.Q., Alexander B.M., Barnholtz-Sloan J.S., Barthel F.P., Batchelor T.T., Bindra R.S., Chang S.M., Chiocca E.A. (2020). Glioblastoma in adults: A Society for Neuro-Oncology (SNO) and European Society of Neuro-Oncology (EANO) consensus review on current management and future directions. Neuro Oncol..

[5] Pouyan A., Ghorbanlo M., Eslami M., Jahanshahi M., Ziaei E., Salami A., Mokhtari K., Shahpasand K., Farahani N., Meybodi T.E. (2025). Glioblastoma multiforme: Insights into pathogenesis, key signaling pathways, and therapeutic strategies. Mol. Cancer.

[6] Wang L.M., Englander Z.K., Miller M.L., Bruce J.N. (2023). Malignant Glioma. Adv. Exp. Med. Biol..

[7] Richardson T.E., Walker J.M., Hambardzumyan D., Brem S., Hatanpaa K.J., Viapiano M.S., Pai B., Umphlett M., Becher O.J., Snuderl M. (2024). Genetic and epigenetic instability as an underlying driver of progression and aggressive behavior in IDH-mutant astrocytoma. Acta Neuropathol..

[8] Arrieta V.A., Dmello C., McGrail D.J., Brat D.J., Lee-Chang C., Heimberger A.B., Chand D., Stupp R., Sonabend A.M. (2023). Immune checkpoint blockade in glioblastoma: From tumor heterogeneity to personalized treatment. J. Clin. Investig..

[9] Yasinjan F., Xing Y., Geng H., Guo R., Yang L., Liu Z., Wang H. (2023). Immunotherapy: A promising approach for glioma treatment. Front. Immunol..

[10] Watowich M.B., Gilbert M.R., Larion M. (2023). T cell exhaustion in malignant gliomas. Trends Cancer.

[11] Bikfalvi A., da Costa C.A., Avril T., Barnier J.V., Bauchet L., Brisson L., Cartron P.F., Castel H., Chevet E., Chneiweiss H. (2023). Challenges in glioblastoma research: Focus on the tumor microenvironment. Trends Cancer.

[12] Staudinger M., Glorius P., Burger R., Kellner C., Klausz K., Gunther A., Repp R., Klapper W., Gramatzki M., Peipp M. (2014). The novel immunotoxin HM1.24-ETA’ induces apoptosis in multiple myeloma cells. Blood Cancer J..

[13] Mahauad-Fernandez W.D., Naushad W., Panzner T.D., Bashir A., Lal G., Okeoma C.M. (2018). BST-2 promotes survival in circulation and pulmonary metastatic seeding of breast cancer cells. Sci. Rep..

[14] Casarrubios M., Provencio M., Nadal E., Insa A., Del Rosario Garcia-Campelo M., Lazaro-Quintela M., Domine M., Majem M., Rodriguez-Abreu D., Martinez-Marti A. (2022). Tumor microenvironment gene expression profiles associated to complete pathological response and disease progression in resectable NSCLC patients treated with neoadjuvant chemoimmunotherapy. J. Immunother. Cancer.

[15] He X., Chen H., Zhong X., Wang Y., Hu Z., Huang H., Zhao S., Wei P., Shi D., Li D. (2023). BST2 induced macrophage M2 polarization to promote the progression of colorectal cancer. Int. J. Biol. Sci..

[16] Sutton M.N., Lu Z., Li Y.C., Zhou Y., Huang T., Reger A.S., Hurwitz A.M., Palzkill T., Logsdon C., Liang X. (2019). DIRAS3 (ARHI) Blocks RAS/MAPK Signaling by Binding Directly to RAS and Disrupting RAS Clusters. Cell Rep..

[17] Bildik G., Liang X., Sutton M.N., Bast R.C., Lu Z. (2022). DIRAS3: An Imprinted Tumor Suppressor Gene that Regulates RAS and PI3K-driven Cancer Growth, Motility, Autophagy, and Tumor Dormancy. Mol. Cancer Ther..

[18] Bildik G., Gray J.P., Mao W., Yang H., Ozyurt R., Orellana V.R., De Wever O., Carey M.S., Bast R.C., Lu Z. (2024). DIRAS3 induces autophagy and enhances sensitivity to anti-autophagic therapy in KRAS-driven pancreatic and ovarian carcinomas. Autophagy.

[19] Lawson K.A., Sousa C.M., Zhang X., Kim E., Akthar R., Caumanns J.J., Yao Y., Mikolajewicz N., Ross C., Brown K.R. (2020). Functional genomic landscape of cancer-intrinsic evasion of killing by T cells. Nature.

[20] Wang Q., Hu B., Hu X., Kim H., Squatrito M., Scarpace L., deCarvalho A.C., Lyu S., Li P., Li Y. (2018). Tumor Evolution of Glioma-Intrinsic Gene Expression Subtypes Associates with Immunological Changes in the Microenvironment. Cancer Cell.

[21] Lah T.T., Novak M., Breznik B. (2020). Brain malignancies: Glioblastoma and brain metastases. Semin. Cancer Biol..

[22] Yan H., Parsons D.W., Jin G., McLendon R., Rasheed B.A., Yuan W., Kos I., Batinic-Haberle I., Jones S., Riggins G.J. (2009). IDH1 and IDH2 mutations in gliomas. N. Engl. J. Med..

[23] Flavahan W.A., Drier Y., Liau B.B., Gillespie S.M., Venteicher A.S., Stemmer-Rachamimov A.O., Suva M.L., Bernstein B.E. (2016). Insulator dysfunction and oncogene activation in IDH mutant gliomas. Nature.

[24] Malta T.M., Sokolov A., Gentles A.J., Burzykowski T., Poisson L., Weinstein J.N., Kaminska B., Huelsken J., Omberg L., Gevaert O. (2018). Machine Learning Identifies Stemness Features Associated with Oncogenic Dedifferentiation. Cell.

[25] Danaher P., Warren S., Dennis L., D’Amico L., White A., Disis M.L., Geller M.A., Odunsi K., Beechem J., Fling S.P. (2017). Gene expression markers of Tumor Infiltrating Leukocytes. J. Immunother. Cancer.

[26] van den Bent M.J., Geurts M., French P.J., Smits M., Capper D., Bromberg J.E.C., Chang S.M. (2023). Primary brain tumours in adults. Lancet.

[27] van Tellingen O., Yetkin-Arik B., de Gooijer M.C., Wesseling P., Wurdinger T., de Vries H.E. (2015). Overcoming the blood-brain tumor barrier for effective glioblastoma treatment. Drug Resist. Updates.

[28] Stepanenko A.A., Sosnovtseva A.O., Valikhov M.P., Chernysheva A.A., Abramova O.V., Pavlov K.A., Chekhonin V.P. (2024). Systemic and local immunosuppression in glioblastoma and its prognostic significance. Front. Immunol..

[29] Yang F., Zhang D., Jiang H., Ye J., Zhang L., Bagley S.J., Winkler J., Gong Y., Fan Y. (2023). Small-molecule toosendanin reverses macrophage-mediated immunosuppression to overcome glioblastoma resistance to immunotherapy. Sci. Transl. Med..

[30] Geraldo L.H., Xu Y., Jacob L., Pibouin-Fragner L., Rao R., Maissa N., Verreault M., Lemaire N., Knosp C., Lesaffre C. (2021). SLIT2/ROBO signaling in tumor-associated microglia and macrophages drives glioblastoma immunosuppression and vascular dysmorphia. J. Clin. Investig..

[31] Khan F., Pang L., Dunterman M., Lesniak M.S., Heimberger A.B., Chen P. (2023). Macrophages and microglia in glioblastoma: Heterogeneity, plasticity, and therapy. J. Clin. Investig..

[32] van Hooren L., Handgraaf S.M., Kloosterman D.J., Karimi E., van Mil L., Gassama A.A., Solsona B.G., de Groot M.H.P., Brandsma D., Quail D.F. (2023). CD103(+) regulatory T cells underlie resistance to radio-immunotherapy and impair CD8(+) T cell activation in glioblastoma. Nat. Cancer.

[33] Read R.D., Tapp Z.M., Rajappa P., Hambardzumyan D. (2024). Glioblastoma microenvironment-from biology to therapy. Genes Dev..

[34] Yu H., Bian Q., Wang X., Wang X., Lai L., Wu Z., Zhao Z., Ban B. (2024). Bone marrow stromal cell antigen 2: Tumor biology, signaling pathway and therapeutic targeting (Review). Oncol. Rep..

[35] Mahauad-Fernandez W.D., DeMali K.A., Olivier A.K., Okeoma C.M. (2014). Bone marrow stromal antigen 2 expressed in cancer cells promotes mammary tumor growth and metastasis. Breast Cancer Res..

[36] Zheng C., Wang J., Zhou Y., Duan Y., Zheng R., Xie Y., Wei X., Wu J., Shen H., Ye M. (2024). IFNalpha-induced BST2^+^ tumor-associated macrophages facilitate immunosuppression and tumor growth in pancreatic cancer by ERK-CXCL7 signaling. Cell Rep..

[37] Shan F., Shen S., Wang X., Chen G. (2023). BST2 regulated by the transcription factor STAT1 can promote metastasis, invasion and proliferation of oral squamous cell carcinoma via the AKT/ERK1/2 signaling pathway. Int. J. Oncol..

[38] Xia H., Green D.R., Zou W. (2021). Autophagy in tumour immunity and therapy. Nat. Rev. Cancer.

[39] Debnath J., Gammoh N., Ryan K.M. (2023). Autophagy and autophagy-related pathways in cancer. Nat. Rev. Mol. Cell Biol..

[40] Lu Z., Baquero M.T., Yang H., Yang M., Reger A.S., Kim C., Levine D.A., Clarke C.H., Liao W.S., Bast R.C. (2014). DIRAS3 regulates the autophagosome initiation complex in dormant ovarian cancer cells. Autophagy.

[41] Mahgoub E., Taneera J., Sulaiman N., Saber-Ayad M. (2022). The role of autophagy in colorectal cancer: Impact on pathogenesis and implications in therapy. Front. Med..

[42] Yamamoto K., Venida A., Yano J., Biancur D.E., Kakiuchi M., Gupta S., Sohn A.S.W., Mukhopadhyay S., Lin E.Y., Parker S.J. (2020). Autophagy promotes immune evasion of pancreatic cancer by degrading MHC-I. Nature.

[43] Herhaus L., Gestal-Mato U., Eapen V.V., Macinkovic I., Bailey H.J., Prieto-Garcia C., Misra M., Jacomin A.C., Ammanath A.V., Bagaric I. (2024). IRGQ-mediated autophagy in MHC class I quality control promotes tumor immune evasion. Cell.

