# Supplementary figures and images for: BST2 and DIRAS3 Drive Immune Evasion and Tumor Progression in High-Grade Glioma
Source: Int J Mol Sci. 2025 Jun 27;26(13):6205. doi: 10.3390/ijms26136205 (PMC12250118; doi:10.3390/ijms26136205)

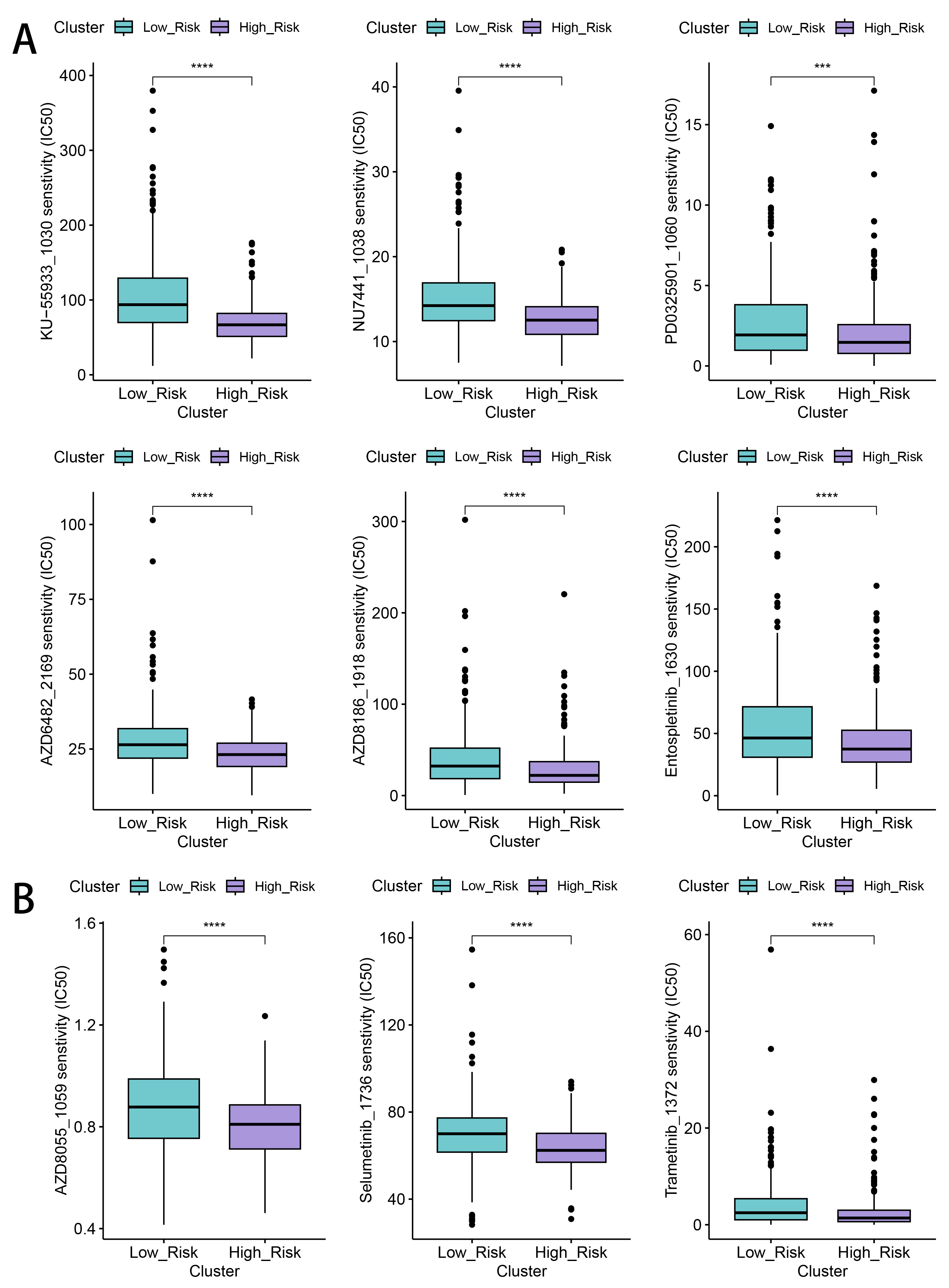

Supplement: Supplementary file 1 [file ijms-26-06205-s001.zip › Figure S2_re.tiff]
